# The waiting room: Unmet sexual health service needs among men and gender-diverse individuals having sex with men in England, findings from an online, cross-sectional community survey in 2024

**DOI:** 10.1177/09564624251413004

**Published:** 2026-01-08

**Authors:** Dana Ogaz, Dolores Mullen, George Baldry, Danielle Jayes, Dawn Phillips, Catherine M Lowndes, David Reid, Jordan Charlesworth, Erna Buitendam, David Phillips, Gwenda Hughes, Catherine H Mercer, John Saunders, Kate Folkard, Katy Sinka, Hamish Mohammed

**Affiliations:** 1 Blood Safety, Hepatitis, STI & HIV Division, 371011 UK Health Security Agency, London, UK; 2 The National Institute for Health and Care Research Health Protection Research Unit in Blood Borne and Sexually Transmitted Infections at University College London in Partnership with the UK Health Security Agency, London, UK; 3 Institute for Global Health, 4919 University College London, London, UK; 4 7885 Croydon University Hospital, Croydon Health Services NHS Trust, London, UK; 5 UK Public Health Rapid Support Team, London School of Hygiene and Tropical Medicine, London, UK

**Keywords:** homosexual, other, other, other, prevention, sexual behaviour

## Abstract

**Background:**

Sexual health service (SHS) delivery in England shifted substantially with rapid expansion of online services during the COVID-19 pandemic. While digital services may improve reach, limited data exist on unmet need for in-person SHS in England, especially among men and gender-diverse individuals who have sex with men, key populations disproportionately affected by sexual health inequalities.

**Methods:**

We analysed data from “Reducing inequalities in Sexual Health” (RiiSH) 2024 (Nov/Dec 2024), an online survey of UK-resident men and gender-diverse individuals having sex with men. We assessed in-person SHS access and unmet need (tried but failed to access a SHS in-person) over the past year. Bivariate and multivariable logistic regression was used to examine associations with unmet need.

**Results:**

Among 2404 participants living in England (median age 45 years, 88% White, 95% cisgender), 86% had ever accessed in-person SHS and 59% in the past year. Of those who tried to access in-person care in the past year, 12% (95% CI: 11%–14%) experienced unmet need, especially Outside London (15% vs 8% in London). Common barriers included unavailable (50%) or inconvenient (41%) appointment times. In adjusted multivariable analysis, unmet need continued to be lower among participants living in London (aOR: 0.64 [95% CI: 0.44–0.92]), those financially comfortable (aOR: 0.69 [0.49–0.97]), and those reporting ≥1 marker(s) of sexual risk (e.g. HIV-PrEP use in the last year and/or in the last 3–4 months, the report of a bacterial STI diagnosis, engaging in chemsex, having had ≥10 male physical sex partners; aOR: 0.14 [0.10–0.20]). Unmet need was higher among participants with limiting long-term physical health conditions (aOR: 1.61 [1.12–2.30]) and those who reported ever using online postal self-sampling services for STI testing (OPSS) (aOR: 1.50 [1.07–2.09]).

**Conclusions:**

Despite high SHS engagement, one-in-eight reported unmet need for in-person SHS. Local service delivery guided by joint strategic needs assessments could help address unmet need for SHS.

## Background

In England, sexual health service delivery is primarily provided through publicly funded services which are free, open access (i.e. without referral from primary care), and confidential. Services include testing and treatment for sexually transmitted infections (STIs) and provision of STI and HIV prevention interventions (e.g. HIV pre-exposure prophylaxis [PrEP], HIV post-exposure prophylaxis [PEP], doxycycline post-exposure prophylaxis [doxyPEP], vaccination, condoms). Access is largely available through a network of specialist sexual health services (SHS) and increasingly via online platforms offering online postal self-sampling kits (OPSS) (i.e. self-collection of samples sent for laboratory testing with results sent to users). While some clinics offer walk-in services, many in-person appointments are dependent on online or telephone triage,^1^ with waiting times varying by local supply and demand.

Over the past decade, the sexual health landscape in England has undergone profound transformation.^2^ Service provision has been significantly impacted by the COVID-19 pandemic, accelerating already-evolving models of service delivery, amidst increasingly constrained public health budgets and clinical resources.^3,4^ The proportion of OPSS testing provided through SHS has rapidly increased, representing 13% of all STI testing in 2019 (pre-COVID-19) to 42% in 2024.^5^ While OPSS has increased the reach of SHS for many,^6–8^ significant structural- and individual-level barriers to use remain. These include digital exclusion, low health literacy, and difficulties with self-sampling (particularly for blood samples), as well as concerns about confidentiality.^9,10^ Service limitations exist on the provision of self-sampling services, such as locally imposed restrictions on eligibility and number of test kits available.^11,12^ Inherent limitations of online interactions^13,14^ that include reduced ability to identify those with increased risk or complex needs, inability to deliver injections (e.g. vaccinations), and obscuring the need for clinicians to provide care for acute and complex conditions^13^ pose significant challenges to comprehensive sexual health service provision.

Previous assessments of unmet need for STI and HIV testing in England have considered the general population,^4^ or gay, bisexual, and other men who have sex with men (GBMSM),^15^ and have largely been based on researcher perceived need and defined on behavioural proxies (e.g. based on clinical history). While important, these assessments do not characterise unmet needs among those attempting to access care or who may already be experiencing SHS access inequalities. Given the changing sexual health landscape, especially following periods of major disruption and service reconfigurations, there is a need to examine in-person SHS accessibility. We use data collected from a large, online community survey to characterise SHS access and unmet need among men and gender-diverse individuals having sex with men, which are key groups more likely to experience sexual health inequalities in England.^5,16,17^

## Methods

### Data collection and recruitment

The ‘Reducing inequalities in Sexual Health’ (RiiSH) 2024 survey is part of a series of yearly, online cross-sectional surveys, assessing the sexual health and well-being of a community sample of men and gender-diverse individuals who have sex with men in the UK. Recruitment in 2024 took place from 18^th^ November-11^th^ December. Survey recruitment was conducted through advertisements on social networking sites (Facebook, Instagram) and geospatial dating platforms (Grindr, Scruff, Jack’d and Recon). Survey methods have been previously reported.^18,19^ In brief, participants eligible to take part included self-identifying men (cisgender or transgender), transgender women or gender-diverse individuals who were assigned male at birth, aged ≥16 years, resident in the UK and reporting sex with a man (cisgender and/or transgender) in the last year. Given differences in the commissioning of sexual healthcare across the four nations of the UK, analyses were restricted to those living in England.

### SHS access

We conducted descriptive analyses to examine in-person (i.e. face-to-face) SHS access (‘never’, ‘in the last year’). Among those with an in-person visit in the last year, we examined reasons for last visit (e.g. for STI testing) and why services were chosen (e.g. ‘I felt comfortable here’) (see Appendix I for question excerpts). Results were stratified by region of residence (London, Outside London), where we hypothesised greater accessibility in London over other areas. While we posited greater variation in urban vs rural areas, these examinations were not possible given lack of granularity in region of residence measures. We also report the proportion who reported that they had ever used OPSS for STI testing, defined as testing via private or public self-sampling services.

### SHS unmet need

Unmet need was defined as those unable to access an in-person SHS among those who tried. We report the proportion of participants with unmet need (%, 95% CI) and reasons for SHS inaccessibility by region of residence (London, Outside London).

### Factors associated with SHS unmet need

We examined factors associated with unmet need using binary logistic regression among those who tried to access a SHS in the last year. We present bivariate and multivariable associations and consider evidence of association where *p* < 0.05. All specified covariates were included in multivariable modelling based on *a priori* consideration (age-group, sexual orientation) and/or associations in previous literature (e.g. disability, sexual risk).^20–22^ These also included: financial stability (prioritised as a measure of deprivation given strong association with poor sexual health^5^ relative to other available measures, including education); markers of sexual risk (see definition below) as a composite measure given the strong correlation between measures of sexual risk behaviours; as well as region of residence (dichotomised as London, Outside London given measure limitations, see above). We considered inclusion of self-report of physical conditions or illnesses lasting or expected to last for 12 months or more but prioritised the report of physical limitations due to these conditions (see definition below) as these could influence in-person accessibility. As an indirect measure of digital literacy, and/or of structural barriers to in-person SHS access, we also included the report of ever having used OPSS for STI testing.

Having markers of sexual risk was defined as those reporting HIV-PrEP use in the last year and/or in the last 3–4 months: the report of a bacterial STI diagnosis, engaging in chemsex (those that had used crystal meth, mephedrone or GHB/GBL), having had ≥10 male physical sex partners, and meeting partners through sex-on-premises venues, public sex environments (i.e. cruising environments), or at private sex parties (herein collectively called ‘venue risk’).

For those reporting a limitation associated with a long-term physical health condition, we created a binary measure (i.e. response of ‘Yes, a little’, ‘Yes, a lot’ vs ‘Not at all’ to the question: “Does your condition or illness reduce your ability to carry out day-to-day activities). Those not reporting a long-term health condition were classified as having no limitations.

Survey data was collected via the Snap Surveys platform (https://www.snapsurveys.com). Data management and analyses were conducted using Stata v17.0 or higher (StataCorp, College Station, TX, USA).

### Ethics statement

Ethical approval for RiiSH 2023 was granted by the UKHSA Research and Ethics Governance Group (REGG; ref: R&D 524). Online consent was collected from participants and there was no incentive offered to participate.

## Results

Of 2758 participants recruited to RiiSH 2024, 2404 were resident in England (87%) and included in analyses (excluded participants included those living in Scotland [n = 194], Wales [n = 100], and Northern Ireland [n = 60]) (Appendix II). The median age of included participants was 45 years (interquartile range: 36–55); most were of White ethnicity (88%), cisgender male (95%), degree-educated (60%) and employed (78%). Nearly one-third resided in London (31%) (Table 1).

**Table 1.** Sociodemographic, clinical and behavioural characteristics of (1) all participants and (2) those who tried to access a sexual health service in the last year.

	All participants n (column %)	Those who tried to access a SHS in the last year^a^ n (column %)
Total	2404 (100.0)	1629 (100.0)
Age at survey completion (years)	45 (interquartile range: 36–55)	45 (interquartile range: 36–54)
Mean age at survey completion (years)	45.4 (standard deviation: 13.2)	45.2 (standard deviation: 12.7)
*Sociodemographic characteristics*		
Age-group (3 categories)		
16–29	290 (12.1)	168 (10.3)
30–44	875 (36.4)	640 (39.3)
45 and over	1239 (51.5)	821 (50.4)
Ethnic group (all categories)		
White	2104 (87.5)	1398 (85.8)
Black	55 (2.3)	44 (2.7)
Asian	134 (5.6)	99 (6.1)
Mixed or other	111 (4.6)	87 (5.4)
Ethnic group (2 categories)		
All other ethnic groups	300 (12.5)	231 (14.2)
White	2104 (87.5)	1398 (85.8)
Gender identity and sex at birth		
All other gender identity groups	110 (4.6)	76 (4.7)
Cisgender male	2294 (95.4)	1553 (95.3)
Sexual orientation		
Gay/homosexual	1940 (80.7)	1366 (83.9)
Bisexual, straight/heterosexual, or another way^b^	464 (19.3)	263 (16.1)
Region of residence (England)		
London	748 (31.1)	570 (35.0)
South East	323 (13.4)	205 (12.6)
South West	194 (8.1)	111 (6.8)
West Midlands	162 (6.7)	100 (6.1)
North West	316 (13.1)	218 (13.4)
North East	89 (3.7)	61 (3.7)
Yorkshire and Humber	189 (7.9)	122 (7.5)
East Midlands	168 (7.0)	97 (6.0)
East of England	206 (8.6)	141 (8.7)
England - unknown	9 (0.4)	4 (0.2)
UK born		
No	548 (22.8)	403 (24.7)
Yes	1856 (77.2)	1226 (75.3)
Educational qualifications		
Below degree-level	968 (40.3)	611 (37.5)
Degree-level or higher	1436 (59.7)	1018 (62.5)
Employment		
Not employed	539 (22.4)	349 (21.4)
Current employment (full/part-time, self-employed)	1865 (77.6)	1280 (78.6)
Comfortable financial situation		
No	1306 (54.3)	883 (54.2)
Yes (top two quartiles)^c^	1098 (45.7)	746 (45.8)
*Clinical and behavioural characteristics*		
HIV status		
Negative/unknown	2140 (89.0)	1408 (86.4)
PLWHIV (tested positive)	264 (11.0)	221 (13.6)
HIV-PrEP use since Dec 2023 (in last year)		
No (includes PLWHIV and those never reported use)	1286 (53.5)	596 (36.6)
Yes	1118 (46.5)	1033 (63.4)
Ever used STI antibiotic post-exposure prophylaxis for the prevention of STIs		
No	2073 (86.2)	1343 (82.4)
Yes	331 (13.8)	286 (17.6)
Ever reported recreational drug use associated with chemsex		
No	2044 (85.0)	1335 (82.0)
Yes	360 (15.0)	294 (18.0)
No. Of male physical sex partners since Aug 2024 (in last 3–4 months)		
No sex/only virtual sex	215 (8.9)	104 (6.4)
1	274 (11.4)	121 (7.4)
2–4	715 (29.7)	431 (26.5)
5–9	519 (21.6)	394 (24.2)
10 or more	681 (28.3)	579 (35.5)
Whether any male physical sex partners were new since Aug 2024 (in last 3–4 months)		
No new partners	539 (22.4)	258 (15.8)
1 or more new partners	1865 (77.6)	1371 (84.2)
Whether had vaginal/anal sex with a woman since Aug 2024 (in last 3–4 months)		
No	2266 (94.3)	1561 (95.8)
Yes	138 (5.7)	68 (4.2)
Venue risk^d^		
No	1595 (66.3)	968 (59.4)
Yes	809 (33.7)	661 (40.6)
Bacterial STI diagnosis since Aug 2024 (in last 3–4 months)		
No	2160 (89.9)	1393 (85.5)
Yes	244 (10.1)	236 (14.5)
Limiting long-term physical health condition		
No	1904 (79.2)	1290 (79.2)
Yes	500 (20.8)	339 (20.8)
Limiting long-term mental health condition		
No	1816 (75.5)	1214 (74.5)
Yes	588 (24.5)	415 (25.5)
Markers of sexual risk (composite)^e^		
No	836 (34.8)	319 (19.6)
Yes	1568 (65.2)	1310 (80.4)
Ever used a private or public OPSS^f^		
No	1460 (60.7)	956 (58.7)
Yes	944 (39.3)	673 (41.3)
Ever visited an in-person SHS		
No	330 (13.7)	46 (2.8)
Yes	2074 (86.3)	1583 (97.2)
Tried to access in-person SHS in last year		
No	775 (32.2)	…
Yes, tried but unsuccessful	202 (8.4)	202 (12.4)
Yes, visited in-person SHS	1427 (59.4)	1427 (87.6)

^a^Includes 4 participants with missing data on region of England residence and were not included in modelling (see Table 2).

^b^Includes those identifying as bisexual, straight, or another way.

^c^Top two quartiles (“I am comfortable“/”I am very comfortable” from the question, “How would you best describe your current financial situation”.

^d^Met male partners through sex-on-premises venues, public sex environments (i.e. cruising environments), or at private sex parties.

^e^Includes reporting of: HIV-PrEP use in the last year (i.e. since Dec 2023), and/or in the last 3-4 months (i.e. since Aug 2024), the report of a bacterial STI diagnosis, engaging in chemsex, having had ≥10 male physical sex partners, and meeting partners through sex-on-premises venues, public sex environments (i.e. cruising environments), or at private sex parties.

^f^Online and postal self-sampling services for STI testing.

PLWHIV: people living with HIV; HIV-PrEP: HIV pre-exposure prophylaxis; STI: sexually transmitted infection; SHS: sexual health service; OPSS: online and postal self-sampling for STI testing; RiiSH: ‘reducing inequalities in sexual health’.

### SHS access

Of all participants, 86% (2074/2404) reported they had ever had an in-person SHS visit (59% [1427/2404] in the last year) and 40% (944/2404) reported ever using a self-sampling service for STI testing. Among those who reported *never* visiting a SHS, most (71% 235/330) also reported they had never used OPSS for STI testing. Of those who *had* ever visited an in-person SHS, 59% (1225/2074) reported that they had never used an OPSS (Table 1).

#### Reasons for last in-person SHS visit

Among those who ever visited a SHS and had done so in the past year (69% 1427/2074), wanting an STI test or a general sexual health check-up (62% 889/1427) and HIV-PrEP access (48% 684/1427) were the most common reasons for visits (Appendix III). Only 11% of all participants reported having had symptoms as a reason for their last visit.

#### Reasons for choosing an in-person SHS

Considering reasons for choice of SHS, close proximity to or ease to travel from home (66% 938/1427) was most commonly reported. Those living in London were less likely to report that their clinic choice was because it was close to home compared to those living Outside London (58% vs 70%) but were more likely to cite proximity to work (24% vs 17%) or because of the service’s reputation (37% vs 16%) as reasons. Excellent staff (50% 719/1427), and services that suited needs (50% 719/1427) were also commonly reported by all as reasons for choosing the last SHS attended (Appendix IV).

### SHS unmet need

Among those who tried to access a SHS in the last year, 12% (95% CI: 11%–14%, 202/1629) could not, with this proportion varying by whether the participant lived in London (8% [6%-11%] vs Outside London, 15% [12%–17%]; Table 1).

#### Reasons for in-person SHS inaccessibility among those with unmet need

Appointment unavailability (50% 102/202) and inconvenient appointment times (41% 82/202) were the most common reasons for inaccessibility (Appendix V). A higher proportion of those reporting unmet need in London reported inconvenient appointment times (46% vs 39%), no appointment availability (56% vs 49%) and waiting too long for an appointment (33% vs 16%) compared to those living Outside London.

### Factors associated with SHS unmet need

All participants who tried to access a SHS in the past year were included in regression models; 4 participants who did not specify region of residence were excluded from analyses (Table 1). In bivariate analysis, we found a lower likelihood of unmet need amongst older age groups (uOR: 0.55 [0.35–0.86] aged ≥45 vs 16–29); those living in London (uOR: 0.54 [0.38–0.76]; those reporting financial comfort (uOR: 0.59 [0.43–0.80]); and those with at least one marker of sexual risk (uOR: 0.14 [0.10–0.19]). There was a higher likelihood of unmet need amongst those who were bisexual, straight, or described themselves in another way (uOR: 1.82 [1.28–2.59] vs gay/homosexual), had a long-term physical health condition that caused limitations in their everyday life (uOR: 2.01 [1.45–2.77]), and those who had ever used a OPSS for STI testing (uOR: 2.01 [1.45–2.77]) (Table 2).

**Table 2.** Sociodemographic, clinical and behavioural characteristics associated with unmet need among those trying to access a sexual health service in the last year.

	uOR (95% CI)	aOR (95% CI)^a^	*p*-value
*Sociodemographic characteristics*			
Age-group (3 categories)			
16–29	1.00 (base)	1.00 (base)	0.493
30–44	0.69 (0.44–1.10)	1.11 (0.66–1.85)	
45 and over	0.55 (0.35–0.86)	0.89 (0.53–1.51)	
Sexual orientation			
Gay/homosexual	1.00 (base)	1.00 (base)	0.057
Bisexual, straight/heterosexual, or another way^b^	1.82 (1.28–2.59)	1.48 (1.00–2.20)	
London resident			
No	1.00 (base)	1.00 (base)	0.014
Yes	0.54 (0.38–0.76|)	0.64 (0.44–0.92)	
Comfortable financial situation			
No	1.00 (base)	1.00 (base)	0.031
Yes (top two quartiles)^c^	0.59 (0.43–0.80)	0.69 (0.49–0.97)	
*Clinical and behavioural characteristics*			
Markers of sexual risk (composite)^d^			
No	1.00 (base)	1.00 (base)	<0.001
Yes	0.14 (0.10–0.19)	0.14 (0.10–0.20)	
Limiting long-term physical health condition			
No	1.00 (base)	1.00 (base)	0.011
Yes	2.01 (1.45–2.77)	1.61 (1.12–2.30)	
Ever used a private or public OPSS^c^			
No	1.00 (base)	1.00 (base)	0.019
Yes	1.06 (0.79–1.43)	1.50 (1.07–2.09)	

^a^Includes 1625 observations; excludes 4 participants that did not specify England region of residence.

^b^Includes those identifying as bisexual, straight, or another way.

^c^Top two quartiles (“I am comfortable”/“I am very comfortable” from the question, “How would you best describe your current financial situation)”. Online and postal self-sampling services for STI testing.

^d^Includes reporting of: HIV-PrEP use in the last year (i.e. since Dec 2023), and/or in the last 3–4 months (i.e. since Aug 2024), the report of a bacterial STI diagnosis, engaging in chemsex, having had ≥10 male physical sex partners, and meeting partners through sex-on-premises venues, public sex environments (i.e. cruising environments), or at private sex parties.

HIV-PrEP: HIV pre-exposure prophylaxis; STI: sexually transmitted infection; SHS: sexual health service; OPSS: online and postal self-sampling for STI testing; RiiSH: ‘reducing inequalities in sexual health’; uOR: unadjusted odds ratio; aOR: adjusted odds ratio.

In a multivariable logistic regression adjusted for age and sexual orientation (*a priori*), as well as region of residence, self-reported financial comfort, markers of sexual risk, limiting long-term physical health conditions and use of OPSS for STI testing, we found no evidence of association by age-group (aOR: 0.89 [0.53–1.51] aged ≥45 vs 16–29) or sexual orientation (aOR: 1.48 [1.00–2.20]). With adjustment, a lower likelihood of unmet need remained amongst London residents (aOR: 0.64 [0.44–0.92]), those financially comfortable (aOR: 0.69 [0.49–0.97]) and those with markers of sexual risk (aOR: 0.14 [0.10–0.20]). Those with a limiting long-term physical health condition (aOR: 1.61 [1.12–2.30]), and those that had ever used an OPSS (aOR: 1.50 [1.07–2.09]) had a higher likelihood of unmet need.

## Discussion

Analysis of this large, online community survey provides important insights into patterns of SHS use and unmet need of men and gender-diverse individuals who have sex with men in England. While most participants had ever accessed in-person SHS, unmet need was evident among those who had recently attempted to access a SHS in-person. Findings emphasise the importance of assessing whether current service models, many of which originate from adaptations made to SHS delivery during the COVID-19 pandemic, adequately meet contemporary needs. Equitable access to SHS will be critical for successful implementation of preventative interventions such as doxyPEP and 4CMenB vaccination as well as ongoing HIV combination prevention strategies in England. Ensuring accessibility will be essential to realising the full potential of emerging and existing preventative tools. Continued evaluation of mixed delivery models (in-person, online) will be needed to maximise SHS effectiveness and reach, especially when addressing symptomatic versus preventative needs.

In-person SHS use was high among participants (86% ever), with a visit in the last year reported among 59% of participants, highlighting the continued demand for in-person care even amid the growing availability of remote SHS delivery such as self-sampling. Having symptoms did not appear to be a major driver of attending SHS in this sample; however, HIV-PrEP access (48%) and STI testing (62%) were both common reasons for seeking an in-person visit. Both reasons may reflect the high uptake of HIV-PrEP among GBMSM in England, and adherence to national recommendations for quarterly STI and HIV testing for individuals having condomless sex with new partners,^23–25^ reinforcing the integral role of SHS for delivering STI preventative care. Only 40% had ever used an OPSS for STI testing, and many who had attended in-person SHS had never used an OPSS, suggesting that online options may not yet be fully accessible or substitutive for all. Understanding the reasons for preferring in-person versus OPSS, including trust, convenience, or additional support, requires further exploration.

Though most RiiSH participants accessed an in-person SHS, 12% of those who tried to access in-person care in the past year reported unmet need, with this proportion significantly higher for those groups known to be more likely to experience health inequalities. Though we found weak evidence of an association (*p* = 0.057), there were indications of higher levels of unmet need reported by participants identifying as bisexual or heterosexual, suggesting ongoing barriers in service design or engagement strategies, particularly in services targeted to self-identifying gay and bisexual men. These findings highlight the need for more inclusive programmes of targeted interventions and a stronger equity lens in SHS provision and commissioning.^26,27^ Financial comfort, living in London, and having at least one marker of sexual risk were independently associated with lower likelihood of unmet SHS need, while unmet need was higher among those with a long-term limiting health condition and those who had previously used OPSS. These findings underscore the complexity of access, which is not just about service availability but, at an individual-level, must also consider capacity and preference to use those services. Our findings echo those from quasi-representative surveys of the general population, which reported a higher likelihood of unmet need among individuals with a limiting long-term condition, a stark reflection of persistent barriers to SHS faced by people living with disabilities.^21,28^

Unmet need also varied substantially by whether participants lived in or Outside London, suggesting that in-person SHS may not equally serve or be equally available to people living in different parts of England. This may also reflect greater SHS availability in London relative to other locations in England, where, by region, London has one of the highest number of SHSs in England (42/247).^29^ Appointment unavailability and inconvenient appointment times were commonly-reported barriers to in-person SHS access, but were reported more frequently by London residents, potentially reflecting system pressure in high-demand services.^2,30^ A mixed methods assessment of the use of OPSS services in the UK found that in-person SHS case-mix complexity increased following the introduction of OPSS.^31^ While the use of online services and lower levels of unmet need among those with markers of sexual risk suggests redirections of clinical complexity that prioritise those with the greatest clinical need, the impacts of this displacement among other potential service users are largely unexplored. Even less is known about the role of SHS as a gateway to broader healthcare in England, including mental health services, vaccination, and substance misuse support.^32,33^ The importance of these touchpoints should be a key consideration in digital health planning, especially as digital services could expand.^34^

Overall, our findings suggest the need for the evaluation of SHS service models, which will require a calibrated balance between in-person and online services in order to meet the needs of men and gender-diverse individuals who have sex with men more broadly. While digital services may increase reach and service capacity,^35,36^ they should not replace in-person care where it is essential (e.g. vaccination) or could be the gateway to wider healthcare provision for vulnerable or marginalised groups who could benefit from further or integrated services.^37^ Barriers to in-person access, where appropriate, must be minimised. Locally informed, equity-focused strategies are essential for addressing these gaps. We posit that joint strategic needs assessments^38^ conducted at local level and used to inform SHS commissioning could act as key levers for targeted interventions that could minimise unmet need. These assessments must consider local demand, structural inequalities, and service capacity^39^ to reduce unmet need among those most at risk of being underserved, including sexual minorities, people with disabilities, and those living in areas with fewer SHS, as identified in this analysis.

### Strengths and limitations

A strength of this study is that it reports data from a large, community-based sample of people living across England. However, this sample is self-selected and may over-represent individuals who are more digitally literate, already engaged in SHS and OPSS use, or more motivated to respond due to recent service access experiences. As such, it may not fully reflect the experiences of broader, more population-representative groups. Self-reported data may also be subject to recall or social desirability bias. While we did capture area of participant residence, this may not be geographically representative. This study did not have the statistical power to consider regional differences, or rural and urban settings, where STI and HIV epidemiology and SHS accessibility varies.^5^ While missing data were limited as most responses were compulsory, larger studies are needed to assess differences in gender and sexual minorities (e.g. transgender people, heterosexual identifying men who have sex with men). Alongside, we have no information on distance to in-person SHS, or on the role of SHS loyalty to specific services where reputation may affect service availability which was more common as a reason for choosing a service among London-based participants. It is unclear whether OPSS were used following in-person inaccessibility. We found a higher likelihood of unmet need among those who had ever used an OPSS. However, due to the cross-sectional nature of this study, it is unclear whether this reflects barriers to in-person services or is an indication of routine STI testing practices among participants, such as regular STI testing (including through OPSS) as part of HIV-PrEP care pathways.^23^

We defined unmet need largely as an expressed need^40^ among those who self-reported that they had tried and been unable to access a SHS in the last year. However, expressed need may not equate to actual or objective need and reflects one of many domains of need. Use of our measure could have variable effects. Unmet need could be underestimated as it does not capture individuals affected by broader individual-level (e.g., lack of knowledge, low health literacy) and structural barriers (e.g. regional SHS and OPSS availability, stigma) that may prevent SHS engagement altogether. At the same time, the use of expressed need could have overestimated unmet need, as participants facing SHS inaccessibility would be more likely to recall and report difficulties.

Service access questions (e.g. reasons for visit, why services were chosen) were based on formative work and cognitive testing^18^ that preceded the COVID-19 pandemic, where online services were less common; however major service provision changes were in place at that time (e.g. HIV-PrEP accessibility, quarterly STI and HIV testing recommendations). Notably, even though this sample likely represents a population highly engaged with SHS, unmet need was still present. This may imply that even greater access challenges could exist for groups with a lower perceived risk of STI/HIV acquisition (e.g. heterosexual women, older age groups) or those who may have other unique barriers to using online services.

### Conclusion

While engagement with SHS was high, unmet need was evident among this highly health literate sample. These findings highlight the challenges of maintaining quality in-person SHS while scaling accessible digital options. Addressing these needs will require a multifaceted approach, including the expansion of accessible online and in-person services, as well at the development of co-designed and co-produced interventions in partnership with communities that are equity-focused and locally tailored.

## Supplemental material


Supplemental material - The waiting room: unmet sexual health service needs among men and gender-diverse individuals having sex with men in England, findings from an online, cross-sectional community survey in 2024
Supplemental material for The waiting room: unmet sexual health service needs among men and gender-diverse individuals having sex with men in England, findings from an online, cross-sectional community survey in 2024 by Dana Ogaz, Dolores Mullen, George Baldry, Danielle Jayes, Dawn Phillips, Catherine M Lowndes, David Reid, Jordan Charlesworth, Erna Buitendam, David Phillips, Gwenda Hughes, Catherine H Mercer, John Saunders, Kate Folkard, Katy Sinka and Hamish Moammed in International Journal of STD & AIDS.

## Data Availability

The data that support the findings of this study are not publicly available to protect participant privacy. However, some aggregate data are available upon reasonable request from the UK Health Security Agency (UKHSA). Requests can be directed to DataAccess@ukhsa.gov.uk.

## References

[1] British Association for Sexual Health and HIV (BASHH) . Standards for the management of STIs 2019. [cited 2025 01 August]. Available from: https://www.bashh.org/resources/81/standards_for_the_management_of_stis/

[2] Local Government Association . Breaking point: securing the future of sexual health services. 2022 Available from: https://www.local.gov.uk/publications/breaking-point-securing-future-sexual-health-services

[3] Dema E Sonnenberg P Gibbs J , et al. How did the COVID-19 pandemic affect access to condoms, chlamydia and HIV testing, and cervical cancer screening at a population level in britain? (Natsal-COVID). Sex Transm Infect 2023; 99(4): 261–267. 35981863 10.1136/sextrans-2022-055516 PMC10313967

[4] Dema E Gibbs J Clifton S , et al. Initial impacts of the COVID-19 pandemic on sexual and reproductive health service use and unmet need in britain: findings from a quasi-representative survey (Natsal-COVID). Lancet Public Health 2022; 7(1): e36–e47. 34995541 10.1016/S2468-2667(21)00253-X PMC8730819

[5] Migchelsen SJ Daahir U Macdonald C , et al. Sexually transmitted infections and screening for chlamydia in England: 2024 report 2025. [cited 2025 01 August]. Available from: https://www.gov.uk/government/statistics/sexually-transmitted-infections-stis-annual-data-tables

[6] Conway A Gibbs J Spence T , et al. ‘It's less traumatic because you're in your own home': exploring trauma-informed care for digital sexual health services - a secondary qualitative data analysis. Sex Transm Infect. 2025 Jun 24:sextrans-2024-056442. doi: 10.1136/sextrans-2024-056442. Epub ahead of print. PMC12911605 40555477

[7] Spence T Griffiths F Ross J . Service user experiences of using internet-based testing for sexually transmitted infections (STIs): a qualitative study. Sex Transm Infect 2024; 100(6): 356–361. 38821875 10.1136/sextrans-2024-056228 PMC11347205

[8] Bailey JV Wayal S Aicken CRH , et al. Interactive digital interventions for prevention of sexually transmitted HIV. AIDS 2021; 35(4): 643–653. 33259345 10.1097/QAD.0000000000002780 PMC7924981

[9] Sumray K Lloyd KC Estcourt CS , et al. Access to, usage and clinic outcomes of, online postal sexually transmitted infection services: a scoping review. Sex Transm Infect 2022; 98(7): 528–535. 35701146 10.1136/sextrans-2021-055376 PMC9613868

[10] Flowers P Vojt G Pothoulaki M , et al. Understanding the barriers and facilitators to using self-sampling packs for sexually transmitted infections and blood-borne viruses: thematic analyses for intervention optimization. Br J Health Psychol 2023; 28(1): 156–173. 35918874 10.1111/bjhp.12617 PMC10086833

[11] SH24. [cited 2025 30 September]. Available from: https://sh24.org.uk/

[12] Sexual health London. 2025 [cited 2025 30 September]. Available from: https://www.shl.uk/

[13] Clarke E Horner PJ Muir P , et al. Assessment of online self-testing and self-sampling service providers for sexually transmitted infections against national standards in the UK in 2020. Sex Transm Infect 2023; 99(1): 14–20. 35414607 10.1136/sextrans-2021-055318 PMC9887362

[14] Habel MA Sullivan P Hall C , et al. Remote health: optimizing the delivery of sexual health care. Sex Transm Dis 2022; 49(11S): S1–S6. 10.1097/OLQ.0000000000001618 PMC10197151 35312660

[15] Brown JR Reid D Howarth AR , et al. Sexual behaviour, STI and HIV testing and testing need among gay, bisexual and other men who have sex with men recruited for online surveys pre/post-COVID-19 restrictions in the UK. Sex Transm Infect 2023; 99(7): 467–473. 36858811 10.1136/sextrans-2022-055689 PMC10715464

[16] Mohammed H Blomquist P Ogaz D , et al. 100 years of STIs in the UK: a review of national surveillance data. Sex Transm Infect 2018; 94(8): 553–558. 29654061 10.1136/sextrans-2017-053273

[17] Mercer CH Prah P Field N , et al. The health and well-being of men who have sex with men (MSM) in britain: evidence from the third national survey of sexual attitudes and lifestyles (Natsal-3). BMC Public Health 2016; 16: 525. 27386950 10.1186/s12889-016-3149-z PMC4936006

[18] Wayal S Reid D Blomquist PB , et al. The acceptability and feasibility of implementing a bio-behavioral enhanced surveillance tool for sexually transmitted infections in England: mixed-methods study. JMIR Public Health Surveill 2018; 4(2): e52. 29728348 10.2196/publichealth.9010 PMC5960042

[19] Howarth AR Saunders J Reid D , et al. ‘stay at home’: exploring the impact of the COVID-19 public health response on sexual behaviour and health service use among men who have sex with men: findings from a large online survey in the UK. Sex Transm Infect 2022; 98(5): 346–352. 34544888 10.1136/sextrans-2021-055039 PMC8457994

[20] Sullivan AK Saunders J Desai M , et al. HIV pre-exposure prophylaxis and its implementation in the PrEP impact trial in England: a pragmatic health technology assessment. Lancet HIV 2023; 10(12): e790–e806. 38040478 10.1016/S2352-3018(23)00256-4 PMC7616873

[21] Holdsworth E Trifonova V Tanton C , et al. Sexual behaviours and sexual health outcomes among young adults with limiting disabilities: findings from third British national survey of sexual attitudes and lifestyles (Natsal-3). BMJ Open 2018; 8(7): e019219. 10.1136/bmjopen-2017-019219 PMC6124606 29980540

[22] Ogaz D Enayat Q Brown JRG , et al. Mpox diagnosis, behavioral risk modification, and vaccination uptake among Gay, bisexual, and other men who have sex with men, United Kingdom, 2022. Emerg Infect Dis 2024; 30(5): 916–925. 38573160 10.3201/eid3005.230676 PMC11060451

[23] British Association for HIV (BHIVA), British Association for Sexual Health and HIV (BASHH) . BHIVA/BASHH guideline on the use of HIV pre-exposure prophylaxis 2025. [cited 2025 01 August]. Available from: https://www.bashh.org/resources/5/hiv_preexposure_prophylaxis_2025

[24] British HIV Association, British Association for Sexual Health and HIV, British Infection Association . British HIV association/british association for sexual health and HIV/british infection Association Adult HIV Testing Guidelines 2020. Available from: https://www.bhiva.org/file/5f68c0dd7aefb/HIV-testing-guidelines-2020.pdf

[25] Clutterbuck D Asboe D Barber T , et al. United Kingdom national guideline on the sexual health care of men who have sex with men. Int J STD AIDS 2016; 2018: 956462417746897. 10.1177/0956462417746897 29334885

[26] Curtis T Bennett K Mcdonagh L , et al. P532 the sexual behaviour and health of heterosexual-identifying men who have sex with men: a systematic review. Sex Transm Infect 2019; 95(Suppl 1): A242.

[27] Curtis T Mercer C Field N , et al. O15.6 ‘if they ask, I will tell them’: attitudes towards accessing sexual healthcare among heterosexual-identifying MSM in England. Sex Transm Infect 2021; 97(Suppl 1): A52.

[28] Sturrock B Dema E Pérez RB , et al. Disability and access to sexual and reproductive health services in the first year of the COVID-19 pandemic in britain: findings from Natsal-COVID wave 2 survey. BASHH annual conference 2024, bournemouth, UK, June 17–19 2024. Int J STD AIDS 2024; 35(1_suppl): 1–157.

[29] UK Health Security Agency . Quality and methodology information and development plan for official statistics in development on sexually transmitted infections. 2025 [cited 2025 08 August]. Available from: https://www.gov.uk/government/statistics/sexually-transmitted-infections-stis-annual-data-tables/quality-and-methodology-information-and-development-plan-for-official-statistics-in-development-on-sexually-transmitted-infections

[30] Waters A . Sexual health services are at “breaking point” after £1bn in cuts since 2015. Br Med J 2022; 379: o2766. 36384672 10.1136/bmj.o2766

[31] Bennett J Howarth A Tostevin A , et al. Assessing the impact of online postal self-sampling on clinical complexity in a sexual health service in England: a mixed methods analysis. BASHH Annual Conference 2025, June 9–11 2025 International Journal of STD & AIDS 2025; 2025(36): 1–156.

[32] Giraudon I Schmidt AJ Mohammed H . Surveillance of sexualised drug use - the challenges and the opportunities. Int J Drug Pol 2018; 55: 149–154. 10.1016/j.drugpo.2018.03.017 29598967

[33] Hibbert MP Germain JS Brett CE , et al. Service provision and barriers to care for men who have sex with men engaging in chemsex and sexualised drug use in England. Int J Drug Pol 2021; 92: 103090. 10.1016/j.drugpo.2020.103090 33513457

[34] Department of Health and Social Care . Local public health services given £200 million boost. 2025 [cited 2025 22 July]. Available from: https://www.gov.uk/government/news/local-public-health-services-given-200-million-boost

[35] Howarth A Harb A Mohammed H , et al. Uptake, positivity, and equity of online postal self-sampling for chlamydia testing in England: a retrospective cohort study. Lancet Reg Health Eur 2025; 56: 101412. 40893446 10.1016/j.lanepe.2025.101412 PMC12392761

[36] Turner KME Zienkiewicz AK Syred J , et al. Web-Based activity within a sexual health economy: observational Study. J Med Internet Res 2018; 20(3): e74. 29514776 10.2196/jmir.8101 PMC5863011

[37] Sewell WC Powell VE Ball-Burack M , et al. Brief report: “I Didn't Really Have a Primary Care Provider Until I Got PrEP”: Pptients' Pepspectives on HIV Prpexposure Prpphylaxis as a Gageway to Hehlth Cace. J Acquir Immune Defic Syndr 2021; 88(1): 31–35. 34397743 10.1097/QAI.0000000000002719 PMC8369038

[38] Department of Health . Statutory guidance on joint strategic needs assessments and joint health and wellbeing strategies 2013. 2025 Available from: https://www.gov.uk/government/publications/jsnas-and-jhws-statutory-guidance

[39] Pulford C Bell E Fagan L , et al. STI prioritisation framework. 2024 [cited 2025 20 February]. Available from: https://www.gov.uk/government/publications/sti-prioritisation-framework

[40] Bradshaw J . A taxonomy of social need. Problems and progress in medical care: essays on Current research, 7th series. Oxford University Press, 1972, pp. 70–82.

