# Supplementary material for: The waiting room: Unmet sexual health service needs among men and gender-diverse individuals having sex with men in England, findings from an online, cross-sectional community survey in 2024
Source: Int J STD AIDS. 2026 Jan 8;37(5):538–47. doi: 10.1177/09564624251413004 (PMC12982575; doi:10.1177/09564624251413004)
Supplement: Supplemental material - The waiting room: unmet sexual health service needs among men and gender-diverse individuals having sex with men in England, findings from an online, cross-sectional community survey in 2024 [file sj-pdf-1-std-10.1177_09564624251413004.pdf]

Supplementary material

Appendix I. RiiSH 2024 question excerpts.. 2

Appendix II. RiiSH 2024 participant flowchart.. 5

Appendix III: Reasons for last in-person SHS among those with a visit in the last year 6

Appendix IV: Reasons for choosing last in-person SHS among those with a visit in the last year 7

Appendix V: Reasons for in-person SHS inaccessibility among those who tried and were unable to access a SHS in the last year.. 8

Appendix I. RiiSH 2024 question excerpts

Excerpt from "About sexual health services" section

Q92. (Compulsory question)

Routing: Asked to all

Have you **ever visited** a sexual health service (i.e. had a **face-to-face appointment**)?

- No
- Yes

Q93. (Compulsory question)

Routing: Asked where Q92=Yes

When was the **last time** you visited a sexual health service?

- Less than one year ago
- One to two years ago
- More than two years ago

Q94. (Compulsory question)

Routing: Asked where Q93=Less than one year ago

Thinking back to your **last** visit to a sexual health service, was it:

- Before August 2024
- Since the start of August 2024

Q95. (Non-compulsory question)

Routing: Asked where Q92=Yes

Why did you attend a sexual health service (**the last time if more than once**)? [*tick all that apply*]

- I wanted an STI test or a general sexual health check-up
- I had no symptoms, but I was worried I might have an STI or HIV
- I had symptoms
- A sexual partner had symptoms
- A sexual partner was diagnosed with an STI
- Treatment after a previous positive test
- Check-up after a previous positive test
- As follow-up to an online test
- Ongoing HIV care and treatment
- To get ***post***-exposure prophylaxis (PEP) to prevent HIV (i.e. taken **after** sex)
- To get ***pre***-exposure prophylaxis (PrEP) to prevent HIV (i.e. taken **before** sex)
- Following sexual assault
- Following domestic violence
- I was told to attend by my GP/family doctor or another healthcare professional

- I needed condoms
- I needed contraception (other than condoms)
- I needed a vaccination
- I couldn't get an online testing kit
- For another reason

****

Excerpt from "About sexual health services" section

Q97. (Compulsory question)

Routing: Asked where Q92=No OR Q93=One to two years ago, More than two years ago

Thinking back to **the last year**, have you tried to get a face-to-face appointment to a sexual health service?

- No, I did not try to get a face-to-face appointment
- Yes, I did try to get a face-to-face appointment

Q98. (Compulsory question)

Routing: Asked where Q97= Yes, I did try to get a face-to-face appointment

In the **last year**, why were you unable to get a face-to-face appointment at a sexual health service? [tick all that apply]

- Unsuitable opening hours
- No appointments at convenient times
- No appointment availability
- I had to wait too long to get an appointment
- I was directed to online services instead
- Difficulty travelling to the appointment
- Work commitments
- Family commitments
- Another reason(s)

Q100. (Compulsory question)

Routing: Where Q93= Less than one year ago

Thinking about your **most recent** face-to-face visit to a sexual health service, please select what were the most important factors in your decision to use this service? [*tick all that apply*]

- The clinic was close by or easy to get to from my home
- The clinic was close by or easy to get to from my workplace
- The services on offer suited my needs very well
- No appointment was needed
- The staff were excellent
- The clinic has a great reputation
- No-one I know would likely have seen me going there so my privacy is protected

- The clinic offers/offered particular services which were important to me
- It was easy to get an appointment
- I felt comfortable here
- I felt involved in decisions in my care
- Staff had time to discuss my needs
- Another reason <free text box>
- Prefer not to say

****

Excerpt from "Testing for sexually transmitted infections (STIs) other than HIV" section

Q60. (Non-compulsory question)

Routing: Asked where Q57=Yes (Q57 not shown, Ever tested for STIs]

Where have you **tested** for STIs other than HIV? *[tick all that apply]*

- At a sexual health service
- I used a free online self-sampling service [I took my own sample and sent it off for the result]
- I used a self-testing kit [taking my own sample and finding out the result immediately]
- At my GP practice
- At a community HIV testing service (that is not in a hospital or clinic)
- At an HIV clinic
- I used a private online self-sampling service (I had to pay for it) [I took my own sample and sent it off for the result]
- At a private medical practice (i.e. I had to pay for the service)
- At a mobile medical unit
- Somewhere else

***

Excerpt from "About your sexual satisfaction and personal well-being" section

Q115. (Non-compulsory question)
Routing: Asked to all

Do you have any physical conditions or illnesses lasting or expected to last for **12 months or more**?

- Yes
- No

Q115a. (Non-compulsory question)
Asked where Q115=Yes
Does your condition or illness / do any of your conditions or illnesses reduce your ability to carry out day-to-day activities?

- Yes, a lot
- Yes, a little
- Not at all

Appendix II. RiiSH 2024 participant flowchart

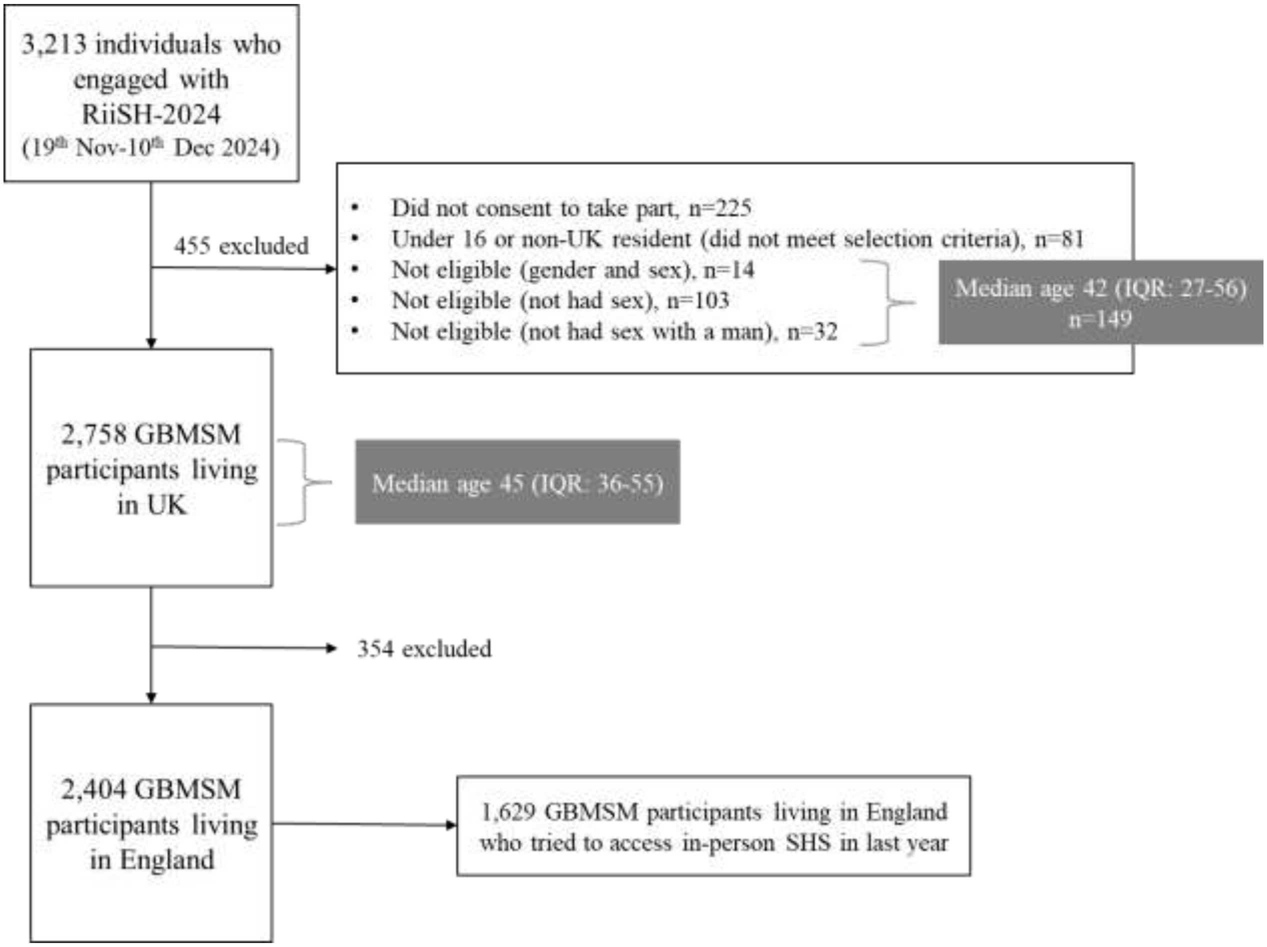

Appendix III: Reasons for last in-person SHS among those with a visit in the last year

	All	London	Outside London
**Reason**	(n=1427)	(n=522)	(n=901)
I wanted an STI test or a general sexual health check-up	62%	62%	62%
I had no symptoms, but I was worried I might have an STI or HIV	10%	9%	11%
I had symptoms	11%	14%	9%
A sexual partner had symptoms	4%	5%	3%
A sexual partner was diagnosed with an STI	7%	7%	6%
Treatment after a previous positive test	7%	7%	6%
Check-up after a previous positive test	4%	3%	5%
As follow-up to an online test	3%	3%	2%
Ongoing HIV care and treatment	10%	10%	10%
To get post-exposure prophylaxis (PEP) to prevent HIV (i.e. taken after sex)	4%	3%	4%
To get pre-exposure prophylaxis (PrEP) to prevent HIV (i.e. taken before sex)	48%	46%	49%
Following sexual assault‡	<1%	<1%	<1%
Following domestic violence‡	<1%	<1%	<1%
I was told to attend by my GP/family doctor or another healthcare professional‡	<1%	<1%	<1%
I needed condoms‡	<2%	<2%	3%
I needed contraception (other than condoms)‡	<1%	<1%	<1%
I needed a vaccination	6%	7%	5%
I couldn’t get an online testing kit‡	<1%	<1%	<1%
Another reason	3%	2%	3%

Responses not mutually exclusive. ‡Proportion masked due to small numbers

Appendix IV: Reasons for choosing last in-person SHS among those with a visit in the last year

Reason	All (n=1427)	London (n=522)	Outside London (n=901)
The clinic was close by or easy to get to from my home	66%	58%	70%
The clinic was close by or easy to get to from my work	19%	24%	17%
The services on offer suited my needs very well	50%	53%	48%
No appointment was needed	7%	9%	6%
The staff were excellent	50%	46%	53%
The clinic has a great reputation	24%	37%	16%
No-one I know would likely have seen me going there so my privacy is protected	7%	6%	8%
offered particular services which were important to me	21%	24%	19%
It was easy to get an appointment	33%	37%	31%
I felt comfortable here	43%	43%	43%
I felt involved in decisions in my care	23%	18%	26%
Staff had time to discuss my needs	35%	30%	38%
Another reason	6%	4%	6%
Prefer not to say‡	<1%	<1%	<1%

Responses not mutually exclusive unless specified. ‡ Exclusive reponse choice.

Appendix V: Reasons for in-person SHS inaccessibility among those who tried and were unable to access a SHS in the last year

Reason	All (n=202)	London (n=48)	Outside London (n=154)
Unsuitable opening hours	29%	33%	28%
No appointments at convenient times	41%	46%	39%
No appointment availability	50%	56%	49%
I had to wait too long to get an appointment	20%	33%	16%
I was directed to online services instead	35%	38%	34%
Difficulty travelling to the appointment	11%	8%	12%
Work commitments	18%	21%	17%
Family commitments	4%	6%	3%
Another reason	9%	8%	10%

Responses not mutually exclusive.
